# Hydrogen Sulfide as Potential Regulatory *Gasotransmitter* in Arthritic Diseases

**DOI:** 10.3390/ijms21041180

**Published:** 2020-02-11

**Authors:** Flavia Sunzini, Susanna De Stefano, Maria Sole Chimenti, Sonia Melino

**Affiliations:** 1 Institute of Infection Immunity and Inflammation, University of Glasgow, 120 University, Glasgow G31 8TA, UK; flavia.sunzini@gmail.com; 2 Rheumatology, Allergology and clinical immunology, University of Rome Tor Vergata, via Montpelier, 00133 Rome, Italy; maria.sole.chimenti@uniroma2.it; 3 Department of Chemical Science and Technologies, University of Rome Tor Vergata, via della Ricerca Scientifica 1, 00133 Rome, Italy; destefanosusanna@gmail.com

**Keywords:** inflammation, arthritis, organosulfur compounds, oxidative stress, stem-cell therapy, H_2_S-releasing biomaterials, non-steroidal anti-inflammatory drugs (NSAIDs)

## Abstract

The social and economic impact of chronic inflammatory diseases, such as arthritis, explains the growing interest of the research in this field. The antioxidant and anti-inflammatory properties of the endogenous *gasotransmitter* hydrogen sulfide (H_2_S) were recently demonstrated in the context of different inflammatory diseases. In particular, H_2_S is able to suppress the production of pro-inflammatory mediations by lymphocytes and innate immunity cells. Considering these biological effects of H_2_S, a potential role in the treatment of inflammatory arthritis, such as rheumatoid arthritis (RA), can be postulated. However, despite the growing interest in H_2_S, more evidence is needed to understand the pathophysiology and the potential of H_2_S as a therapeutic agent. Within this review, we provide an overview on H_2_S biological effects, on its role in immune-mediated inflammatory diseases, on H_2_S releasing drugs, and on systems of tissue repair and regeneration that are currently under investigation for potential therapeutic applications in arthritic diseases.

## 1. Introduction

Hydrogen sulfide is an endogenously produced biological agent belonging to the *gasotransmitter* family. The physiological role and the relevance of this molecule are rapidly expanding. Endogenous H_2_S plays pivotal roles in the biochemical pathways of the central nervous, respiratory, and cardiovascular systems. This *gasotransmitter* is physiologically present in the human body, and it is mainly produced endogenously by four enzymes: cystathionine beta-synthase (CBS EC 4.2.1.22), cystathionine gamma-lyase (CSE, EC 4.4.1.1), 3-mercaptopyruvate sulfotransferase (MST, EC 2.8.1.2), and cysteine aminotransferase (CAT) (reviewed in references [1,2,3,4,5]). However, other enzymes such as thiosulfate sulfurtransferase (TST) [6] and the more recently discovered selenium-binding protein 1 (SELENBP1) are able to catalyze H_2_S production [7].

Although there are limits of measurement techniques and the quantification of biologic H_2_S levels is debated, H_2_S physiological levels may range from 50–160 μM in the mammalian brain to 30 nM–100 μM in the peripheral blood and 25 μM in the synovial fluid of patients with non-inflammatory arthritis [8,9,10,11,12,13]. It is known that a relevant fraction of H_2_S is bound to proteins in several tissues, such as hemoglobin [14,15]. An endogenous source of H_2_S is also represented by the enterobacterial flora and by the non-enzymatic reduction of sulfurs [4].

**Figure 1:**
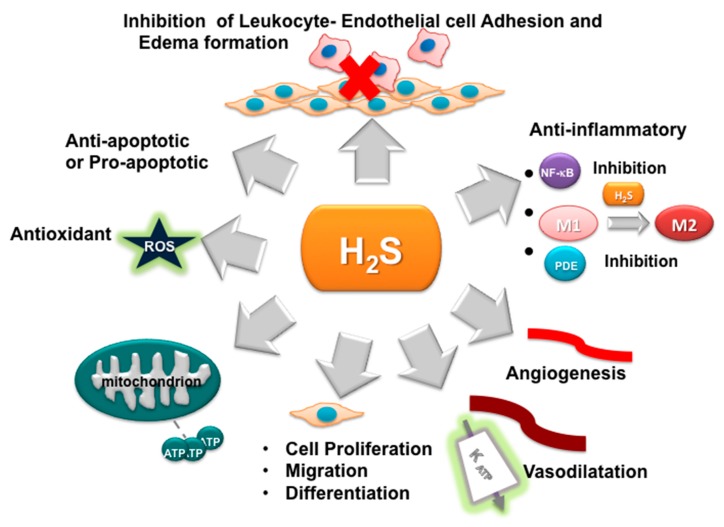
Schematic description of the effects of H_2_S. The anti-inflammatory effect of H_2_S is due to its ability to inhibit some essential pro-inflammatory transcription factors and intracellular signaling, such as nuclear factor κB (NF-κB) and phosphodiesterases (PDEs), and to improve angiogenesis through K_ATP_ channel/mitogen-activated protein kinase (MAPK) pathway activation. It inhibits the production of inflammatory cytokines and avoids the adhesion of leukocytes and endothelial cells. Moreover, the *gasotransmitter* can have pro- or anti-apoptotic effects depending on the cell type and its concentration. At the appropriate concentration, it is also able to have an anti-apoptotic effect due to its antioxidant properties, as well as its ability to increase the mitochondrial activity and the expression of anti-apoptotic proteins [17]. However, exogenous H_2_S is also able to induce apoptosis in cancer cells. H_2_S can also act on the vascular smooth muscle producing vasodilation. M1, macrophages M1; M2, macrophages M2; K_ATP_, ATP-dependent K -channels.

As a *gasotransmitter*, H_2_S can pass freely through cell membranes and does not require a specific receptor to mediate its effect. Only recently, H_2_S was considered an important signaling molecule. The biological effects of H_2_S are multiple and opposite, depending on its concentration. The first H_2_S biological effect was discovered in the vascular system, with the ability to induce the relaxation of vascular smooth muscle, causing vasodilation [16]. Despite the controversial role initially attributed to H_2_S, it is to date recognized that, at low concentrations, it exhibits anti-apoptotic, anti-nociceptive, cardio-protective, and blood pressure-lowering effects, while also improving angiogenesis, via the activation of K_ATP_ channels and extracellular signal-regulated kinases, such as Akt pathways [1,17,18,19,20,21,22]. Moreover, H_2_S shows neuroprotective and anti-inflammatory effects in general due to its antioxidant effects and inhibition of pro-inflammatory cytokines [23,24,25,26,27,28]. The potential effects of H_2_S were also discussed in several review articles [29,30,31,32] and are here summarized in Figure 1.

In the last few years, many studies demonstrated a relevant role of H_2_S to mediate the inflammation and the processes of tissues repair. Chronic inflammation is the key feature of inflammatory arthritis, such as rheumatoid arthritis (RA) and psoriatic arthritis (PsA). The impact of chronic inflammatory diseases on the quality of life and autonomy in the daily activities in a wide population with different ages and the consequent high economic costs explain the growing interest in this field. Recently, the connection between H_2_S and joint inflammation, in the context of arthritis, is growing, either as a pathogenic or potential therapeutic role. Therefore, in this review, we describe the effects of H_2_S on inflammatory arthritis and its potential therapeutic approach. 

## 2. Oxidative Stress and Inflammation in Arthritis

**Figure 2:**
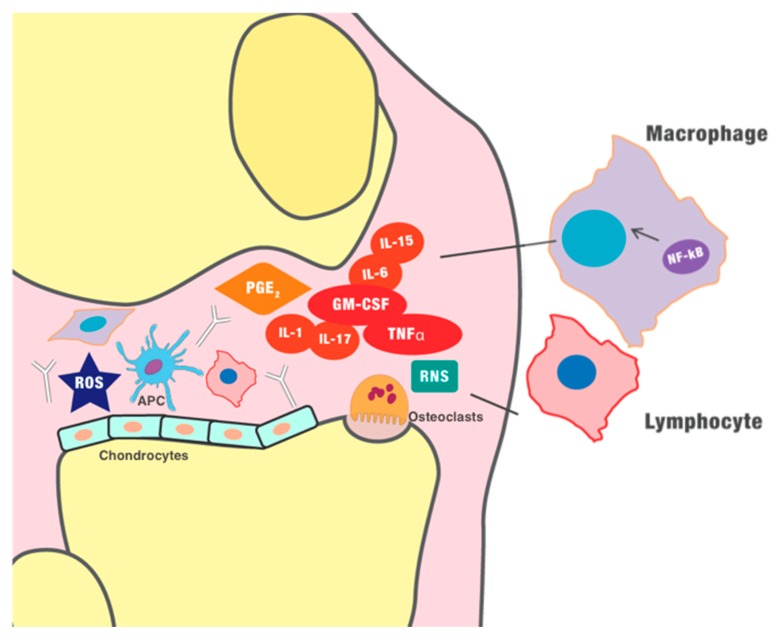
Pathogenesis of inflammatory arthritides. Self-reactive T helper cells seem involved in the maintenance of inflammation, further sustained by B cells, especially in rheumatoid arthritis (RA), where it is possible to detect autoantibodies (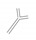), such as the rheumatoid factor and anti-cyclic citrullinated peptide (anti-CCP) antibodies. Furthermore, innate immunity is involved in chronic inflammation. Granulocyte-macrophage colony-stimulating factor (GM-CSF) induces the differentiation of the macrophages. Monocytes and macrophages, as well as dendritic cells, seem to play a vital role in the pathogenesis of inflammatory arthritis, as antigen-presenting cells (APCs), and they can express several pro-inflammatory cytokines. In early stages of inflammation, hypoxia and the production of ROS (reactive oxygen species) and RNS (reactive nitrogen species) seems to play a role in the initiation of the inflammatory process and induction of angiogenesis. The new blood vessels further maximize the recruitment of immune cells, amplifying the inflammatory process. The chronic inflammation finally perpetuates the production of pro-inflammatory cytokines (such as tumor necrosis factor-α (TNF-α)) and other mediators, such as prostaglandin E2, which ultimately generate vasodilation, infiltration of immune cells, and destruction of the cartilage. 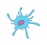, dendritic cell/APC; 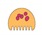, osteoclast; 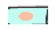, chondrocyte.

RA and PsA represent the most common chronic inflammatory arthritis. They share common pathogenic features, both linked to chronic inflammation secondary to a dysregulation of the immune response; however, the clinical manifestations and outcomes are usually different. Despite the clinical and pathological differences, both diseases share some similarities in the inflammatory pathways. In the course of active arthritis, joint inflammation is overall characterized by increased vascularization, oxidative stress, and infiltration of immune cells in the synovium (Figure 2); these events induce fibroblast-like synoviocyte (FLS) hypertrophy which, ultimately, perpetuates the inflammation, generating a chronic loop. In the early stages of arthritis, the oxidative stress seems to have a key role in initiating the inflammatory process, as demonstrated in some studies [33,34]. Moreover, oxidative stress may account for post-translational modifications of proteins, potentially responsible for autoreactive antibody production [35], particular to RA. Synovial angiogenesis is also an early alteration in the arthritic joint and is characterized by endothelial swelling, cell infiltration, and tortuous vessels. The amplified expression in the synovium of pro-inflammatory cytokines and growth factors, particularly vascular endothelial growth factor (VEGF), contributes to increased vascularity [36]. However, the new vessels are mostly dysfunctional and, consequently, the RA/PsA synovial membrane results hypoxic. Unsurprisingly, the resulting synovial hypoxia was correlated with local joint inflammation [37]. In the inflamed synovium, altered mitochondrial function and oxidative damage were also observed, which are both possibly related to hypoxia [38,39]. The perpetuation of inflammation leads to damage of the cartilage, which allows the invasion of the subchondral bone by FLS, immune cells, and pro-inflammatory cytokines [40]. The exposure of the subchondral bone to the action of proteinase and activation of osteoclasts (OCs) leads to the characteristic bony erosions (Figure 2). Additionally, PsA is also characterized by inflammation of the enthesis associated with a peculiar osteoproductive phenomenon, which leads to calcification of tendons, ankylosis of joints, and consequently to impaired quality of life. This phenomenon is possibly related to impaired mechanisms of tissue repair. Despite the recent progress and advances, the pathogenic mechanisms behind RA and PsA onset are far from being completely understood. The pathogenesis of inflammatory arthritis is characterized by an immune dysregulation, which involves the activation of both innate and adaptive immunity. Immune-mediated pathogenesis was demonstrated by different evidence, such as the presence of auto-reactive T cells in the synovium [41], the association with major histocompatibility complex (MHC)-I, and the good response to immunosuppressive drugs [42]. An altered metabolic response of the immune cells may also contribute to the perpetuation of the inflammatory loop in the inflamed synovium. FLS and synovial macrophages are enhancers of joint inflammation, which ultimately leads to cartilage disruption [43]. In fact, FLS have a tumor-like behaviour in PsA joints. They are characterized by a few key features, i.e., the increased proliferation and invasiveness, the resistance to apoptosis, and the active production of matrix-degrading enzymes and pro-inflammatory cytokines, such as tumor necrosis factor-α (TNF-α), and interleukin 17 (IL-17) [44]. Regarding innate immunity, dendritic cells (DCs) are present in the synovium, synovial fluid, and ectopic lymph tissue of RA inflamed joints compared with osteoarthritis; in the context of inflammation, DCs have a role as antigen-presenting cells (APCs) and as a producer of pro-inflammatory cytokines, i.e., IL-23 and IL-12, which induce the differentiation of T helper 17 cells (Th17) and Th1 subsets, relevant in joint inflammation [45]. Moreover, defective regulatory T cells are associated with autoimmune disorders, such as RA [46]. Furthermore, macrophages play an active role in the pathogenesis of inflammatory arthritides because of the high expression of pro-inflammatory cytokines and matrix metalloproteinases, and they are APCs for T cells and B cells, representing a source of osteoclast precursors in an inflammatory context [47]. Lymphocytes infiltrate the synovium, where aggregates of T cells and B cells are found, and their absence is associated with remission [48,49]. Despite the progress in arthritis treatment over the last few decades, up to 40% of patients are still non-responders to the available treatments; for this reason, research toward a better understanding of the pathogenesis is continuously growing with a particular interest in new pathogenic mechanisms and possible therapeutic targets.

## 3. H_2_S as Inhibitor of Oxidative Stress and Inflammation

**Figure 3:**
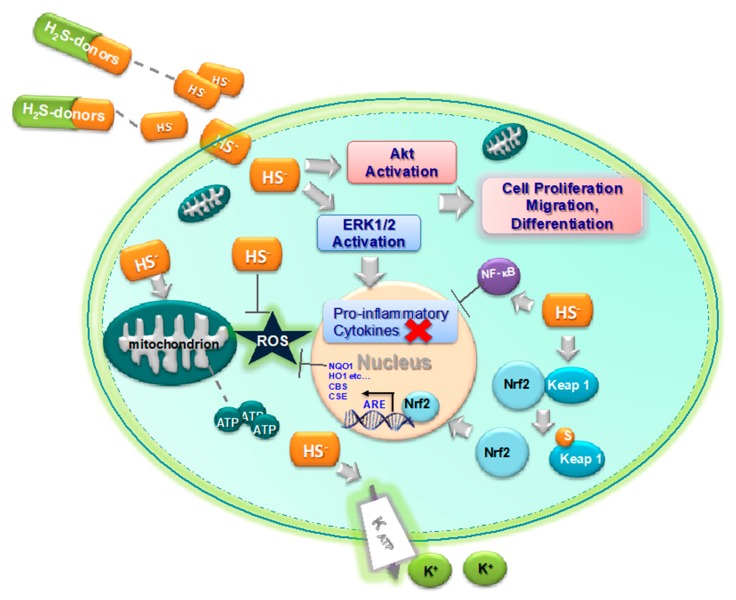
Biological anti-inflammatory effects of H_2_S. H_2_S exerts an anti-inflammatory effect via different biologic effects: direct and indirect reducing action (Nrf2, ARE activation), a regulatory effect on the immune system via NF-kB interaction, interference with rolling and migration of circulating cells, inhibition of enzymes involved in the inflammatory signaling (protein tyrosine phosphatases (PTPs), PDEs). H_2_S induces separation between Nrf2 and Keap1, allowing Nrf2 to enter the nucleus and bind to the ARE gene; furthermore, it modulates in a dose-dependent manner the expression of many cytokine genes, while it obtains an anti-apoptotic effect through Akt activation. H_2_S, through action on ATP-sensitive potassium channels (K_ATP_), inhibits the expression of adhesion molecules on the leukocytes (cluster of differentiation (CD)11/CD18) and endothelium (P-selectin, intracellular adhesion molecule 1 (ICAM1)).

H_2_S can be an endogenous mediator to limit free radical damage and inflammation [50]. A relevant role is played by H_2_S in balancing oxidative and reductive species, thus influencing the cell’s redox state. H_2_S is a strong reducing agent able to directly react with multiple oxidant stressors including superoxide radical anion [51], hydrogen peroxide [52], and peroxynitrite (ONOO^−^) [50] (see Figure 3). Furthermore, H_2_S is able to antagonize lipid peroxidation and oxidation of thiols, to reverse mitochondrial dysfunction [53], and to increase the activity of the most important enzymes involved in the cell’s antioxidant defense. One of these enzymes, the Cu/Zn superoxide dismutase (SOD) [54], is a target of H_2_S, which binds to the catalytic Cu center, thus increasing the ROS scavenging activity. Moreover, the persulfuration of Cys-111 of the human SOD1 stabilizes the enzyme against oxidation-induced aggregation without affecting its activity [55]. The activity of other enzymes, also implicated in the cell’s antioxidant response, such as catalase (CAT), glutathione reductase (GR), glutathione *S*-transferase (GST), quinone reductase (QR), and glutathione peroxidase (GPx), was likewise augmented in rat kidney upon treatment with diallyl sulfide (DAS), which is an H_2_S-releasing molecule [56].

H_2_S is also able to upregulate the antioxidant response elements (ARE) gene transcription [57] (Figure 3) and to produce glutathione persulfide (GSSH) in mitochondria [50,52], a more efficient H_2_O_2_-scavenging molecule than GSH. In more detail, H_2_S induces the dissociation between nuclear erythroid factor 2-related factor 2 (Nrf2) and Kelch-like ECH-associated protein 1 (Keap1) through the sulfhydration of Keap 1 at the Cys-151 residue and induction of a disulfide bond between Cys-288 and Cys-613 residues, thus allowing the Nrf2 nuclear translocation and binding to AREs (Figure 3) [58,59,60,61,62,63]. The antioxidant activity of this *gasotransmitter* is also related to the activation of K_ATP_ channels to reduce oxidative glutamate toxicity [64]. Moreover, the anti-oxidative effects of H_2_S are also related to the anti-inflammatory effect via an increase in the expression of anti-oxidant enzymes, such as indoleamine-pyrrole 2,3-dioxygenase 1 (IDO1) and heme oxygenase 1 (HO1), SOD, CAT, and GPx, which lead to the suppression of reactive oxygen species (ROS) production (Figure 3) [65,66]. Surely, the direct antioxidant effect as a free radical scavenger and as an inducer of antioxidant enzymes might have a potential anti-inflammatory effect in joint inflammation [67,68].

Interestingly, H_2_S is also an inflammation modulator that can have both pro- and anti-inflammatory effects on immune cells, depending on the concentration [68]. Commonly, a pro-inflammatory effect was observed at high H_2_S concentrations, whereas an anti-inflammatory effect was observed at physiological concentrations. Overall, the role of H_2_S in resolving ongoing inflammation and inducing tissue repair was suggested [26,31,69,70,71]. The exogenous administration of H_2_S at physiological levels enhances T-cell activation in T-cell lines and upregulates the expression of cluster of differentiation 69 (CD69), IL-2, and CD25 [72]; H_2_S-induced signaling plays a key role in T-cell activation. Moreover, H_2_S shows a regulatory interaction with IL-10. Furthermore, the upregulation of IL-10 expression was observed after the exogenous administration of H_2_S [73]. The ability of H_2_S donors to modulate the expression of genes for many pro-inflammatory cytokines, chemokines, and enzymes is largely linked to effects on NF-κB activity. In rodent models of colitis, treatment with H_2_S-donors significantly reduced tissue expression of IL-1β, interferon-γ, TNF-α, IL-12 p40, IL-2, regulated upon activation, normal T cell expressed and presumably secreted (RANTES), and inducible nitric oxide synthase (iNOS), without affecting the IL-10 expression [71,74,75]. Allyl disulfide treatment significantly inhibited NF-κB activation and production of TNF-α, as observed by biopsies from patients with ulcerative colitis. It was shown that H_2_S donors are able to reduce TNF-α release following lipopolysaccharide (LPS) exposure in RAW 264.7 cells, a murine monocyte/macrophage-like lineage [76]. Moreover, it was recently demonstrated that H_2_S can shift the macrophage phenotype from pro- to anti-inflammatory [70,77].

Additionally, H_2_S plays also a relevant role in orchestrating immune cell tissue recruitment and infiltration, which are vital in the generation of the inflammatory processes. Leukocyte recruitment and tissue infiltration at the site of inflammation are initial events in inflammation response that are linked to the increase of the production of vascular cell adhesion molecule 1 (VCAM1) and intracellular adhesion molecule 1 (ICAM1) in endothelial cells. H_2_S donors and non-selective inhibitors of CSE and CBS specifically inhibit the migration of leukocytes by directly reducing the adherence of circulating cells to the inflamed vascular walls (as shown in Figure 1); consequently, H_2_S reduces the infiltration of neutrophils and lymphocytes in tissue [78]. H_2_S downregulates ICAM expression in high-glucose-treated [79] and TNF-treated human umbilical vein endothelial cells [80]. Moreover, the upregulation of heme oxygenase 1 (HO1) and inhibition of the NF-κB pathway due to H_2_S-donors can induce the inhibition of VCAM1 expression [81,82]. In neutrophils, H_2_S may also induce the apoptosis, amplifying the anti-inflammatory effect [83].

Moreover, several enzymes involved in the inflammatory response can be inhibited by H_2_S. The majority of protein tyrosine phosphatases (PTPs) have a conserved catalytic domain that contains a cysteine residue, which is able to perform a nucleophilic attack on a substrate; this catalytic residue can be sulfhydrated, as well as in the case of PTP1B. PTP1B is ubiquitously expressed [84] and plays a regulatory role in the control of immune cell signaling in macrophages, monocytes, and granulocytes [85]. PTP1B is important in the release of inflammatory cytokines such as IL-4, IL-6, TNF-*α*, extracellular signal-regulated kinase (ERK), protein kinase B (PKB/Akt) (see Figure 3), human epidermal growth factor receptor 2 (HER2), and NF-κB [84,86,87]. Although PTP1B is a negative regulator of inflammation able to regulate inflammatory processes [88,89], it was also demonstrated that the administration of an inhibitor of PTP1B attenuates the LPS-induced neuroinflammation in mice [90]. PTP1B was the first phosphatase that was shown to be sulfhydrated [91]. The PTP1B sulfhydration occurs at the Cys-215 residue in the active site and leads to inhibition of the PTP activity at a second-order rate (22.4–1.8 M^−1^∙s^−1^) with a rate of H_2_S-mediated PTP1B inactivation of 10–1.4 M^−1^∙s^−1^. In a model of endoplasmic reticulum stress, the sulfhydration of PTP1B decreases its activity, increasing the phosphorylation and activation of eIF2a kinase protein kinase RNA-like endoplasmic reticulum kinase (PERK) Tyr-619, which is a direct PTP1B substrate and has an important role in the endoplasmic reticulum stress response. 

Furthermore, H_2_S can have an inhibitory effect on some phosphodiesterase (PDE) enzymes and, consequently, a positive effect on inflammation [92,93]. PDE inhibitors can have beneficial effects in inflammation by increasing cAMP level, as well as inhibiting the production of ROS and cytokines such as TNF-α and IL-1. Furthermore, PDE5 inhibitors, which increase cGMP levels, can inhibit the production of IL-6 and TNF-α [94,95]. Both endogenous and exogenous H_2_S serves as an inhibitor of PDEs [96]. With a half maximal inhibitory concentration (IC_50_) of 1.6 µM, H_2_S can inhibit the activity of the cGMP-specific PDE5 widely distributed in the cardiovascular system [97,98] and of the mitochondrial PDE2A stimulating mitochondrial electron transport [99]. However, the H_2_S selectivity among different PDE isoforms should be deeply investigated. Notably, the inhibitor of PDE-4, apremilast, was approved for the treatment of PsA [100]; this further highlights the possible therapeutic role of H_2_S in inflammatory arthritis, due to its effect on PDE enzymes. 

The effects on the gastrointestinal tract are representative of the behaviour of this *gasotransmitter* in inflammation. In detail, H_2_S is able to promote healing of experimentally induced stomach ulcers in rats, while treatment with dl-propargylglycine (PAG), which is a CSE inhibitor, has the opposite effect [18,101]. Moreover, ABT-346, which is H_2_S-releasing naproxen, showed significantly less or even the absence of gastrointestinal toxicity compared to naproxen in rats [102].

In the last few years, the molecular mechanism of the H_2_S effect on inflammation was widely investigated. IκB/NF-κB (inhibitor of **κB** /Nuclear factor κ B) is an important molecular pathway that is a key target of H_2_S. NaHS inhibits IκB-α degradation and, therefore, reduces NF-κΒ translocation into the nucleus [103,104]. The first evidence was that LPS-induced NF-κΒ activation in cultured mouse macrophages was inhibited by H_2_S [105]. Subsequently, other studies showed evidence that, in different cell lines, H_2_S regulates the transcription, via NF-κΒ downregulation, of a plethora of pro-inflammatory mediators such as TNF-α, IL-1β, IL-6, IL-8, IL-18, inducible nitric oxide synthase (iNOS), cyclooxygenase-2 (COX-2), and some adhesion molecules. Additionally, slow-releasing H_2_S donors such as GYY4137 (morpholin-4-ium 4 methoxyphenyl (morpholino) phosphinodithioate) *S*-diclofenac, and *S*-propargyl-cysteine (SPRC) have similar effects [106,107,108,109]. Furthermore, H_2_S can also promote cell survival via sulfhydration of the p65 subunit of NF-κB at Cys-38 leading to an anti-apoptotic effect of NF-κB in response to pro-inflammatory agents such as TNF-α and LPS in macrophages [110]. Although the effect of H_2_S on NF-kB is relevant in the inflammation, other transduction mechanisms such as Akt/PKB and MAPK pathways, signal transducer and activator of transcription 3 (STAT-3), and Nrf2 cannot be ruled out as mediator of the inflammatory response to this *gasotransmitter* [56,60,106,111]. Additionally, it was recently demonstrated that H_2_S can induce the activation of forkhead box P3 (FOXP3) and, consequently, the differentiation of T regulatory cells. The functional enhancement of T regulatory cells by H_2_S highlights the role that this T-cell population may have in the H_2_S regulatory network of autoimmune inflammation, i.e., inflammatory arthritis [112].

Interestingly, the stage of inflammation is also relevant for the effect of H_2_S-releasing donors that can be either pro- or anti-inflammatory.

In addition, lower plasma/serum H_2_S concentrations were also related to vascular inflammation associated with childhood disorder Kawasaki disease (autoimmune blood vessel inflammation) [113] and skin inflammation associated with psoriasis [114].

More studies are necessary to not only fully understand the complex role of H_2_S in inflammation, but also to investigate further opportunities for the treatment of existing inflammatory diseases.

## 4. H_2_S and Arthritic Diseases

The effect of H_2_S on joint inflammation was investigated in the last few years due to the anti-inflammatory effect of the *gasotransmitter*. The beneficial effect of H_2_S seems to be dose-dependent; in fact, different studies demonstrated opposite results. At low concentration, H_2_S exerts an anti-inflammatory effect on tissue and cells, suggesting a potentially positive effect on arthritis. The effect of H_2_S on the monocyte/macrophages compartment [69,76] seems particularly relevant due to the central role that macrophages play in the pathogenesis of inflammatory arthritis. Pro-inflammatory macrophages are vital APCs and can differentiate into osteoclasts, cells responsible for irreversible bony erosions; furthermore, macrophages are one of the major sources of TNF-α.

In murine macrophages stimulated with LPS, H_2_S at low concentration inhibits the activation and the synthesis of several pro-inflammatory mediators, i.e., NO, NF-κB, IL-6, and IL-1β; however, at higher concentration, H_2_S stimulates the production of pro-inflammatory molecules by macrophages [107]. This dual effect was also demonstrated on RA FLS by Kloesch et al. [115] in two different experimental stings, showing that short-term exposure to H_2_S induces IL-6 expression, while long-term exposure has the opposite effect [115,116]. Interestingly, in RA synovial fluids, levels of H_2_S were found to be higher than in patients with osteoarthritis (OA); furthermore, the levels detected were positively correlated with disease activity and inflammation. In addition, H_2_S concentration in the bloodstream was increased in patients with RA, and it was also associated with high numbers of circulating leukocytes [13]. As further support of a possible pathogenic role in inflammation, d-penicillamine, used in the past for the treatment of RA, resulted as a direct inhibitor of the synthesis of H_2_S and as a direct inhibitor of CSE activity [117]. In particular circumstances, the pro-inflammatory effect of H_2_S seems to be mediated by the induction of the expression of the intracellular adhesion molecule (ICAM)1, which may increase cell migration into the inflamed tissues [118].

However, H_2_S was found elevated in inflammatory models, and it can represent an attempt at increasing synthesis, to reduce the local inflammation. Various studies demonstrated numerous anti-inflammatory properties of H_2_S mainly explained by the reduced expression of pro-inflammatory mediators and adhesion molecules. In animal models, the administration of H_2_S donors reduced the leukocyte adherence and infiltration and repressed carrageenan-induced paw edema. 

The inhibition of endogenous H_2_S synthesis had the opposite effect, enhancing leukocyte migration [118]. Notably, chondrocytes and mesenchymal stem cells which differentiate in chondrocytes express CBS and CSE, responsible for the synthesis of H_2_S, most probably used as a preserving mechanism [119]. H_2_S showed a protective effect on cartilage damage in OA patients in different studies [120,121]. This effect seems mediated by the inhibition of matrix metalloproteinases and the production of extracellular matrix proteins induced by H_2_S-releasing compounds, which reverted the catabolic effect of IL-1β [122,123]. In addition, H_2_S was demonstrated to reduce the IL-1β-induced expression of IL-6, IL-8, MMP-2, and MMP-14 in OA FLS [124]. Remarkably, H_2_S can also inhibit TNF-α activity, an essential cytokine in inflammatory arthritis pathogenesis, by binding the zinc-proteinase TNF-α-converting enzyme (TACE), as demonstrated by Li et al. [121]. This inhibitory activity may account for the potential anti-inflammatory effect of H_2_S in inflammatory arthritis. Moreover, the NF-κB pathway was inhibited by H_2_S in different experimental studies [117,125]. Hence, the anti-inflammatory role is highly probably secondary to the inhibition of the transcription factor NF-κB, which has a key role in the pathogenesis of inflammatory arthritis as described above (see Figure 2 and Figure 3) [126,127]; furthermore, it can induce the synthesis of the anti-inflammatory cytokine IL-10 [106]. The inhibition of NF-κB activity by H_2_S is, therefore, able to reduce the production of the pro-inflammatory cytokines, mediating the anti-inflammatory effect of the gas in an animal model of sepsis [128], however it may also play a role in joint inflammation. Moreover, NF-κB is a key factor also for the differentiation and maturation of osteoclasts, responsible for bone erosions in arthritis [119]. Interestingly, H_2_S conjugated to the non-steroidal anti-inflammatory drug (NSAID) diclofenac inhibited mature osteoclasts and osteoclastogenesis, thus preventing osteolysis in an animal model of breast cancer metastasis; the inhibitory effect was demonstrated to be dependent on IκB kinase (IKK)/NF-κB [129,130]. This result is particularly interesting, because it may represent a relevant added value to drugs currently used for inflammatory arthritis, not only because H_2_S has a pleiotropic anti-inflammatory profile, but also because it may potentially act on bone erosion, the main long-term target in the treatment of arthritis. Moreover, as described above, H_2_S can directly inhibit the migration and adhesion of leukocytes to endothelial cells (see Figure 1) and the infiltration of neutrophils and lymphocytes [78]. Therefore, defective H_2_S compensatory production can contribute to the pathogenesis of joint inflammation in immune-mediated arthritis.

## 5. H_2_S-Donors as Potential Anti-Arthritis Drugs

H_2_S-donors acquired great therapeutic potential for widely diffused pathologies, such as cardiovascular [131,132], neurodegenerative [133,134,135,136], and gastrointestinal diseases [137,138]. Their H_2_S release can also be prolonged and potentiated by biological thiols that are normally present in biological systems such as protein thiol groups, cysteine, and glutathione (GSH). Furthermore, one of the speculated mechanisms is that H_2_S donors can induce the synthesis of glutathione by increasing the metabolic pathways and enzymes leading to its production [139].

**Figure 4:**
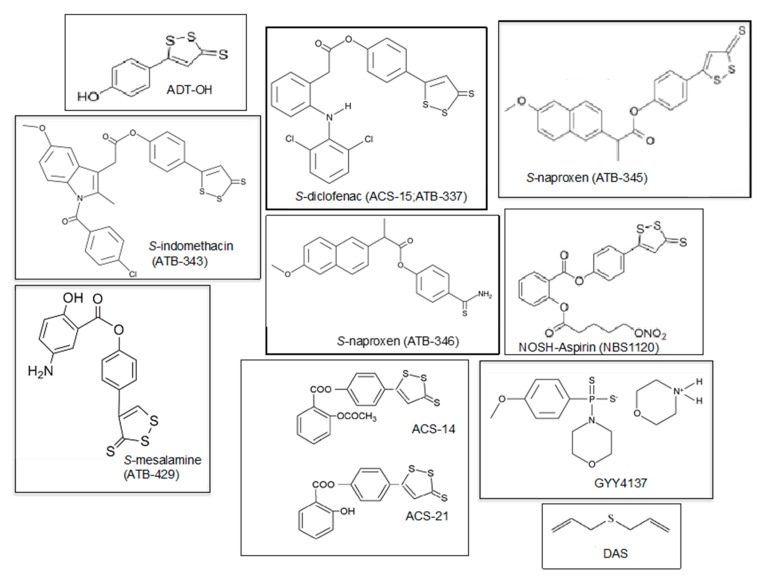
Molecular structures of slow H_2_S-releasing agents with potential anti-inflammatory properties for the treatment of arthritis. ADT-OH (5-(4-hydroxyphenyl)-3*H*-1,2-dithiole-3-thione) and ATB-429 ([4-(5-sulfanylidenedithiol-3-yl) phenyl] 5-amino-2-hydroxybenzoate) are H_2_S-releasing derivatives of mesalamine. ATB-337, ATB- 343, and ATB-345 are respectively diclofenac, indomethacin, and naproxen linked to a hydrogen sulfide-releasing moiety. ATB-346 is naproxen covalently linked to 4-hydroxythiobenzamide (TBZ). ACS-14 is aspirin linked to H_2_S donors, ACS-21 is deacetylated aspirin linked to H_2_S donors, and NBS1120 is a NO H_2_S-releasing derivative of aspirin. GYY4137—morpholin-4-ium 4 methoxyphenyl phosphinodithioate; DAS—diallyl sulfide.

As discussed above, recent evidence of the anti-inflammatory properties and the tissue repair effects of H_2_S increased the interest in its therapeutic potential in arthritis. The research on human species was developed mainly for OA patients, but that on inflammatory arthritis is also promising. Several non-steroidal anti-inflammatory drugs (NSAIDs) were conjugated with H_2_S (Table 1), allowing a slow release of the *gasotransmitter* into the target tissues. The molecular structures of H_2_S donors that are under study for clinical applications are shown in Figure 4. In animal models, NSAIDs conjugated to H_2_S, i.e., naproxen and celecoxib, demonstrated a strong protective effect on gastrointestinal epithelium compared to the toxic effect of the parent drug [102]. For example, the H_2_S-releasing naproxen, called ATB-346, which releases H_2_S via a hydrolytic mechanism [102], was demonstrated to have a greater anti-inflammatory and chondro-protective effect on osteoarthritic joints in animal models, reducing leukocyte migration and reducing TNF-α and NF-κB expression, and less gastrointestinal toxicity [102,140,141]. Recently, a phase II clinical trial investigating 244 healthy subjects demonstrated a drastic reduction of gastric ulcer investigated with endoscopy when treated with ATB-346 (42.2% vs. 2.5% ulcer development with naproxen and ATB-346, respectively). This effect was associated with an increased suppression of COX activity [141]. The efficacy of ATB-346 was recently evaluated in patients with OA, demonstrating that ATB-346 can reduce joint pain, possibly to a greater extent than other standard NSAIDs, such as naproxen or celecoxib [142]. Another derivative drug-releasing H_2_S is *S*-mesalamine (ATB-429) (Figure 4), used for the treatment of inflammatory colitis. ATB-429 exerted a protective role to the gastrointestinal mucosa and higher anti-inflammatory properties than the parent drug [75]. Therefore, ATB-429 could be a good candidate for reducing inflammation in arthritis. Moreover, the synthesis of other compounds able to release both NO and H_2_S recently led to the development of new potential drugs for the treatment of inflammatory arthritis, such as NOSH (nitric oxide and hydrogen sulfide)- sulindac (AVT-18A) and NOSH-aspirin (NBS-1120) (see Table 1). NOSH-sulindac was similar to sulindac in inhibiting the inflammatory response and demonstrating safety in the carrageenan-induced arthritis animal model, but with a larger effect on the reduction of circulating TNF-α level [143]. Similarly, NBS-1120 had a better safety profile in an animal model of systemic and local inflammation when compared to aspirin [144].

Another potential candidate drug for clinical trials in inflammatory diseases is the slow-releasing H_2_S donor, GYY4137, as discussed above. This compound was demonstrated to directly inhibit joint inflammation in a mouse animal model by reducing the production of pro-inflammatory cytokines in macrophages [107]. In particular, GYY4137 inhibits several inflammatory molecules such as IL-1β, IL-6, and TNF-α, in LPS-challenged macrophages in culture [107]; moreover, GYY4137 is able to reduce LPS-evoked septic shock [106] and knee-joint edema in response to intra-articular injection of Freund’s adjuvant [122]. The administration of GYY4137 leads to an anti-inflammatory effect on knee-joint swelling and might be used for clinical applications [82].

Moreover, the endogenous metabolism of natural organosulfur compounds (OSCs) derived from garlic can also lead to the slow-releasing production of H_2_S [126,145] and, consequently, to an antioxidant and anti-inflammatory action [146] with organ/tissue protection. Therefore, OSCs can be considered as potential natural drugs for arthritis. The effects of diallyl sulfide (DAS) on arthritis were investigated in a model of crystal-induced arthritis, human synoviocytes and chondrocytes. DAS was able to inhibit the inflammatory response both ex vivo and in rat with induced joint inflammation [147]. In a recent study, the efficacy of diallyl disulfide (DADS) in controlling joint inflammation was evaluated in an animal model of Freund’s adjuvant-induced arthritis. DADS was demonstrated to be effective in reducing paw edema and joint and cartilage destruction [148]. These studies highlight the therapeutic potential of natural H_2_S donors for the treatment of inflammatory arthritis; however, more in vivo studies are needed to confirm the efficacy of these natural H_2_S donors, their safety profile, and their potential application in inflammatory arthritis.

## 6. Tissue Regeneration as a Therapeutic Approach in Arthritis

The chronic inflammatory process of arthritis leads to the disruption of the cartilaginous tissue, which ultimately contributes to subchondral bone erosions, articular deformities, and impaired quality of life. Physiologically, the cartilage tissue is avascular, aneural, and hypocellular; therefore, there is no self-reparation. A potential therapeutic approach for arthritis is to facilitate its regeneration and/or repair. Linked to the above-cited properties of H_2_S, H_2_S donors could be used as biochemical factors able to induce cartilage and bone repair in the context of arthritis. Therefore, the possibility of fabricating systems conditioned with H_2_S-releasing agents able to improve the repair/regeneration of cartilage and bone in degenerative diseases, as well as RA, is currently being investigated. 

Two main approaches able to improve tissue repair and regeneration were investigated: the injection of stem cells (scaffold-less approach) and of three-dimensional (3D) scaffolds. In the scaffold-less approach, mesenchymal stem cells (MSCs) are the most used kind of cells showing a multipotent property [152,153]. MSCs, under appropriate differentiation stimuli, are able to express chondrogenic potential and improve the repair of cartilage [152,154,155]. 

Intriguingly, the MSC-based approach has the potential to solve some symptoms related to osteoarticular loss; however, it presents various problems such as cell senescence, de-differentiation, and expression of the hypertrophic phenotype. Some of these problems could be solved using the pre-conditioning of MSCs with H_2_S donors. Recently, it was demonstrated that the pretreatment of MSCs with H_2_S provided protection of MSCs upon exposure to hypoxia–ischemic insult whether in vitro or in vivo [156].

The lack of mechanical support in joint repair strategies led to relatively poor results in the clinical application of the scaffold-less approach. Consequently, substances and polymeric supports (both natural or synthetic) were designed with the aim of providing a scaffolding structure and, finally, promoting tissue regeneration more efficiently.

Many synthetic polymers with chondrogenic properties were studied, such as polylactic acid (PLA), polyethylene glycol (PEG), and polycaprolactone (PCL), but only some of them are now commercially available for clinical use [152,157,158,159].

One of these is a poly (l-lactide) (PLLA) scaffold with fibrin gel [157], available in trade under the name *PLA-based*, is made of a porous microstructure with fibrin which results in an excellent combination of mechanical stability, resistance to mechanical stress, and retention of the cellular component. The high level of hydration of the gel is an important requirement for having a homogeneous cellular distribution and excellent values of cell viability.

Injectable gels, based on alginate and hyaluronic acid (HA–MA) were produced to induce cartilage repair [160,161]. Alginate hydrogel showed a highly organized structure with uniform pores, which demonstrated the stimulation of a gradual increase in the cell population and an increase in type II collagen production, which is the principal ECM component of articular cartilage, even if a downregulation of collagen X was observed [160]. The polymerization of this material was obtained directly in situ using an argon laser at 512 nm, and its mechanical properties were easily modulated by the chemical composition. 

Another PEG-based hydrogel scaffold with three-layer composition was also synthetized [158], showing significant effects on cartilage repair. In this scaffold, chondroitin sulfate (CS) and matrix metalloproteinase-sensitive peptides (MMP-pep) were in the upper layer, while CS was in the middle layer, and hyaluronic acid was in the lower layer. This multi-layer composition mimicked the structure of native articular cartilage with mechanical and biochemical properties that change in space. Furthermore, it was seen that this structural variability can stimulate tissue regeneration, leading to an increased production of both collagen II and X.

Another cell-free product that had excellent results for cartilage repair is Gelrin C, a hydrogel scaffold made of PEG–fibrinogen [162,163]. It was demonstrated to effectively induce cartilage repair measured with the Magnetic Resonance Observation of Cartilage Repair Tissue (MOCART) score (84.4 out of 100 MOCART score after 24 months). At the moment, Gelrin C is in clinical phase II for cartilage repair. 

All these biomaterials can be functionalized with H_2_S-donors in order to improve their potential in tissue repair and regeneration. PEG–fibrinogen was, in fact, functionalized by embedding albumin microbubbles able to catalyze the production of H_2_S [164]. This functionalization improved the proliferation of human cardiac progenitor stem cells, promoting their spindled morphology and suggesting a potential application in repair for other biological tissues, such as human cartilage. H_2_S-releasing biomaterials can have several protective actions, including antioxidant and anti-inflammatory effects, angiogenesis, and vasodilation, thus improving the regenerative capacity of polymers. At the moment, there are few in vitro and in vivo studies on scaffolds able to release H_2_S. Scaffolds based on PCL and PLA were produced using an electrospinning technique, and they were respectively functionalized with *N*-benzoyl thio-benzamide (NSHD1) and phosphonamidothioate templates that generate H_2_S in a pH-controlled manner, as well as slow-releasing H_2_S donors extracted from garlic (GaOS and DADS) [159,165,166]. These three scaffolds showed protective effects on the oxidative damage of ROS and the ability to improve cell viability. A sponge H_2_S-releasing silk fibroin (SF) was also doped with GYY4137 [167], resulting in a scaffold with the same mechanical properties, which was able to induce a significant increase in differentiation to mature osteoblasts (OBs) and expression of osteogenic genes after three weeks of growth in a 3D culture of human MSCs. Table 2 summarizes scaffolds and H_2_S-releasing scaffolds that are currently under investigation for tissue repair. The H_2_S-releasing scaffolds could have a potential therapeutic application in the degenerative stages of inflammatory arthritis.

## 7. Conclusions

Currently, the interest in the pathological and therapeutic role of H_2_S in inflammatory diseases is growing. Recently, the evidence of the anti-inflammatory properties of H_2_S at physiological concentrations increased. As a *gasotransmitter,* H_2_S is involved in the regulation of production and release of several cytokines, as well as in the differentiation of adaptative and innate immune cells. Moreover, H_2_S plays a role as a radical scavenger in hypoxic conditions, frequently associated with inflammatory arthritis, and as a promoter of tissue repair.

Due to the growing evidence, several therapeutic approaches were investigated in different inflammatory diseases. In the context of inflammatory arthritis, the most interesting approaches result in the conjugation of anti-inflammatory compounds with H_2_S and the induction of tissue repair. 

The first therapeutic approach aims to target the early stages of the inflammatory process or active inflammation. Despite the recent advances in the approach to inflammatory joint diseases, a significant number of patients need multi-therapeutic strategies with not always successfully outcomes. In this context, the use of H_2_S-conjugated drugs may be a potential add-on treatment. NSAIDs conjugated with H_2_S were already demonstrated to be effective in managing pain in OA patients with significantly reduced toxicity.

The modern treatments for inflammatory arthritis are able to target the active inflammation; however, strategies to treat established bone and cartilage damage are currently lacking. Scaffolds and H_2_S-functionalized scaffolds, with or without cell deliveries, are opening a completely new therapeutic approach in the arthritis field. Currently, a surgical approach is the most used in advanced cases; however, it can have its limitations in elderly patients and with contraindications, and this treatment is not always indicated in patients with mild joint damage. The possibility to induce tissue repair in a damaged joint with the associated anti-inflammatory effect of H_2_S represents a very promising new potential approach. H_2_S-functionalized scaffolds are still in early clinical studies as most of the studies applied them in vitro or in animal models. It will be promising to combine injectable hydrogel scaffolds that stimulate cartilage repair, such as Gelrin C (Regentis, Haifa, Israel), which is in a phase II clinical trial for early OA, with H_2_S-donors and MSCs for further potential clinical applications in both OA and inflammatory arthritis.

Clearly, more work is needed to improve the sensitivity and specificity of H_2_S assays, as well as to improve the patient selection in studies assessing the efficacy of this new promising treatment strategy.

## Figures and Tables

**Table 1:** H_2_S-releasing drugs as potential anti-inflammatory drugs in arthritis.

H_2_S-Derivative Drug	Drug	Company	Clinical Phase	Clinical Applications	References
AVT-18A	Sulindac	Sulfidris	Preclinical	Cancer, inflammation	[143]
NBS-1120	Aspirin		Preclinical	Cancer, inflammation	[144]
ACS-14	Aspirin	CTG Ph.	Preclinical	Inflammation, cardiac injury, Arthritis	[149]
ACS-21	Aspirin	CTG Ph.	Preclinical	Inflammation, cardiac injury, Osteoarthritis	[149]
ACS-6	Ketorolac	CTG Ph.	Preclinical	Arthritis Antioxidant	[149,150]
ATB-337/ACS-15	Diclofenac	Antibe T.	Preclinical	Arthritis, inflammation	[149]
ATB-343	Naproxen	Antibe T.	Preclinical	Inflammatory diseases, Alzheimer’s disease	[149]
ATB-346	Naproxen	Antibe T.	Phase II	Osteoarthritis, inflammation	[102,141,149]
ATB-345	Naproxen	Antibe T.	Preclinical	Inflammatory diseases	[102]
ATB-429	Meselamine	Antibe T.	Preclinical	Cancer, inflammatory diseases, colitis	[75]
GYY4137 DAS/DADS		National Uni. of Singapore	Preclinical Preclinical	Inflammatory diseases, cancer, hypertension, arthritis Cancer, arthritis	[82,107,122,151] [147,148]

**Table 2:** Scaffolds and H_2_S-releasing scaffolds for potential applications in the therapy of arthritis.

Scaffold	Characteristics and Effects	Type of Cells	Commercial Product	Phase of Study	References
**PLLA/fibrin** **^1^PLLA/chondrocyte/atelocollagen**	Improved cell proliferation and expression of type I and type II collagen	Chondrocytes	PLA-based BioSeedR-C (BioTissue, AG, Zurich, Switzerland)	In vitro	[157]
**PEG dyacrylate systems** **PEG/chitosan** **PEG/albumin**	In situ photopolymerization and potential modulation of its mechanical properties, increasing of the expression of type I and II collagen and the amount of sulfated GAG	MSCs		In vitro	[158]
**Alginate**	Increase in chondrocyte viability	Chondrocytes		In vivo (SCID mice)	[160]
**Hyaluronic acid/fibrin** **Hyaluronic acid/collagen type I**	In situ photopolymerization, potential modulation of its mechanical properties, stimulation of ECM production and proteoglycan synthesis, and improved chondrocyte growth	Chondrocytes	Hyaluronic-based HyalograftR C autograft (Anika Therapeutics, Inc., Bedford, MA, USA)	In vivo (human)	[161]
**PEG-DA/denatured human fibrinogen (DHF)**	In situ photopolymerization, potential modulation of its mechanical properties, gradual resorption by the body being replaced by new cartilage tissue	Cell free	GelrinC (Regentis, Haifa, Israel)	Phase II	[163,164]
***H_2_S-releasing scaffolds***					
**PCL/NSHD1**	Significant decrease in apoptosis in a model of tissue transplantation, protection from ROS damage, and increase in expression of collagen type I and type III	3T3		In vivo	[154]
**PFM/GaOS or DADS**	Improved MSC proliferation and anti-microbial activity and protective effect against oxidative damage	hMSCs		In vitro	[166]
**TSTMBs-PFHy**	In situ photopolymerization, potential modulation of its mechanical properties, induced spindled morphology of cells and cell proliferation	HFFs hCPCs		In vitro	[164]
**ALG-CHO/2-aminopyridine-5-thiocarboxamide/tetraaniline**	Increase in ejection fraction value, reduction of the myocardial infarct size in rats	ADSCs		In vivo	[168]
**SATO/CaCl_2_**	Decrease in intimal hyperplasia in human veins	Endothelial cells		In vivo	[169]
**SF/GYY4137**	Significant increase in osteogenic differentiation of stem cells, upregulation of osteogenic and angiogenic genes and integrins	OBs, hMSC		In vitro	[167]
**HA or PCL/JK1**	H_2_S release in pH-dependent manner, improved cell proliferation. tissue regeneration, re-epithelialization, collagen deposition, angiogenesis	Raw 264.7		In vivo (mouse Male C57BL)	[165]

^1^PLLA—poly (l-lactide); PEG—polyethylene glycol; PCL—polycaprolactone; NSHD1—N-benzoyl thio-benzamide; GaOS—garlic oil soluble extracts; DADS—diallyl disulfide; SF—silk fibroin; HA—hyaluronic acid; ECM—extracellular matrix; MSC—mesenchymal stem cell; HFF—human foreskin fibroblasts; CPC—cardiac progenitor cell; ADSC—adipose-derived stem cell; OB—osteoblast. TSTMBs-PFHy— fibrinogen hydrogel incorporating albumin microbubbles functionalized with thiosulfate:cyanide sulfurtransferase; ALG-CHO—Partially Oxidized Alginate; PFM poly(lactic) acid fibrous; SATO aromatic peptide amphiphile and the H_2_S moiety, S-aroylthiooxime; PEG-DA—polyethylene glycol diacrylate; JK1—H_2_S donor synthesized from phenylphosphonothioic dichloride.

## Abbreviations

ADSCs	adipose-derived stem cells
ADT-OH	5-(4-hydroxyphenyl)-3H-1:2-dithiole-3-thione
ATB-429	4-(5-sulfanylidenedithiol-3-yl) phenyl 5-amino-2-hydroxybenzoate
ALG-CHO	partially oxidized alginate
anti CCP	anti-cyclic citrullinated peptide
ARE	antioxidant response element
APCs	antigen-presenting cells
bFGF	basic fibroblast growth factor
cAMP	cyclic adenosine monophosphate
CAT	cysteine aminotransferase
CAT	catalase
CD	cluster of differentiation
cGMP	cyclic guanosine monophosphate
COX 2	cyclooxygenase 2
CS	chondroitin sulfate
CBS	cystathionine beta-synthase
CSE	cystathionine gamma lyase
DAS	diallyl sulfide
DCs	dendritic cells
EC	endothelial cells
ECM	extracellular matrix
eIF2a	eukaryotic translation initiation factor 2
ErK	extracellular signal-regulated kinase
FLS	fibroblast like synoviocytes
FOXP3	forkhead box P3
GaOS	garlic oil soluble extracts
GM-CSF	granulocyte macrophage colony stimulating factor
GPx	glutathione peroxidase
GR	glutathione reductase
GSH	glutathione
GSSH	glutathione persulfide
GST	glutathione-S-transferase
HA	hyaluronic acid
hCPCs	human cardiac progenitor cells
HER 2	human epidermal growth factor receptor 2
HFFs	human foreskin fibroblasts
HO1	heme oxygenase 1
ICAM 1	intercellular Adhesion Molecule 1
IDO 1	indoleamine-pyrrole 2:3- dioxygenase 1
IGF 1	insulin-like growth factor 1
IκB/NF-κB	inhibitor of κB /Nuclear factor κ B
IKK	IκB kinase
IL	interleukin
iNOS	inducible nitric oxide synthase
JK1	H_2_S donors synthesized from phenylphosphonothioic dichloride
Keap 1	Kelch-like ECH-associated protein 1
LPS	lipopolysaccharides
M1	macrophages M1
M2	macrophages M2
MAPK	mitogen-activated protein kinase
MBs-PFHy	fibrinogen hydrogel incorporating albumin microbubbles
MHC-I	major histocompatibility complex
MMP	matrix metalloproteinase
MMP	pepmatrix metalloproteinase-sensitive peptides
MOCART	magnetic resonance observation of cartilage repair tissue
MSCs	mesenchymal stem cells
MST	3-mercaptopyruvate sulfotransferase
NFκB	nuclear factor kappa beta
NOSH	nitric oxide and hydrogen sulfide
Nrf2	nuclear erythroid factor 2-related factor 2
NSHD 1	N-benzoyl thio-benzamide
OA	osteoarthritis
OBs	osteoblast cell
OCs	osteoclasts cell
OSC	organosulfur compounds
PAG	propargylglycine
PCL	polycaprolactone
PDE	phosphodiesterases
PEG	polyethylene glycol
PERK	protein kinase RNA-like endoplasmic reticulum kinase
PFM	poly(lactic) acid fibrous
PKB	protein kinase B
PLA	polylactic acid
PLLA	poly (l-lactide)
PsA	psoriatic arthritis
PTPs	protein tyrosine phosphatases
QR	quinone reductase
RA	rheumatoid arthritis
RANTES	regulated upon activation, normal T Cell expressed and presumably secreted
RAW 264.7	murine monocyte/macrophage-like lineage
RNS	reactive nitrogen species
ROS	reactive oxygen species
SATO	S-aroylthiooxime
SELENBP1	selenium-binding protein 1
SF	silk fibroin
SOD	superoxide dismutase
SpA	spondyloarthritis
STAT 3	signal transducer and activator of transcription 3
TBZ	4-hydroxythiobenzamide
TGF-β1	transforming growth factor beta 1
Th	T helper cells
TNF α	tumor necrosis factor-α
TST	thiosulfate sulfurtransferase
VCAM 1	vascular cell adhesion molecule 1
