# Correction: Sunzini, F., et al. Hydrogen Sulfide as a Potential Regulatory *Gasotransmitter* in Arthritic Diseases. *Int. J. Mol. Sci.* 2020, *21*, 1180; doi:10.3390/ijms21041180

**DOI:** 10.3390/ijms21176134

**Published:** 2020-08-25

**Authors:** Flavia Sunzini, Susanna De Stefano, Maria Sole Chimenti, Sonia Melino

**Affiliations:** 1 Institute of Infection Immunity and Inflammation, University of Glasgow, 120 University, Glasgow G31 8TA, UK; flavia.sunzini@gmail.com; 2 Rheumatology, Allergology and clinical immunology, University of Rome Tor Vergata, via Montpelier, 00133 Rome, Italy; maria.sole.chimenti@uniroma2.it; 3 Department of Chemical Science and Technologies, University of Rome Tor Vergata, via della Ricerca Scientifica 1, 00133 Rome, Italy; destefanosusanna@gmail.com

The authors wish to make the following correction to this paper [1]. The authors are sorry to have reported on Page 11 in Table 1 that AVT-18A and NBS-1120 are being developed by Sulfidris Company; this is incorrect. These drugs are being developed by Avicenna Therapeutics. 

Consequently, the authors wish to make, at this time, the following corrections to Table 1:

The authors would like to apologize for any inconvenience caused to the readers by these changes. These changes have no material impact on the conclusions of our paper. We apologize to our readers.

## Figures and Tables

**Table 1:** H_2_S-releasing drugs as potential anti-inflammatory drugs in arthritis.

H_2_S-Derivative Drug	Drug	Company	Clinical Phase	Clinical Applications	References
AVT-18A	Sulindac	Avicenna T.	Preclinical	Cancer, inflammation	[144]
NBS-1120	Aspirin	Avicenna T.	Preclinical	Cancer, inflammation	[145]
ACS-14	Aspirin	CTG Ph.	Preclinical	Inflammation, cardiac injury, Arthritis	[150]
ACS-21	Aspirin	CTG Ph.	Preclinical	Inflammation, cardiac injury, Osteoarthritis	[150]
ACS-6	Ketorolac	CTG Ph.	Preclinical	Arthritis Antioxidant	[150,151]
ATB-337/ACS-15	Diclofenac	Antibe T.	Preclinical	Arthritis, inflammation	[150]
ATB-343	Naproxen	Antibe T.	Preclinical	Inflammatory diseases, Alzheimer’s disease	[150]
ATB-346	Naproxen	Antibe T.	Phase II	Osteoarthritis, inflammation	[102,142,150]
ATB-345	Naproxen	Antibe T.	Preclinical	Inflammatory diseases	[102]
ATB-429	Meselamine	Antibe T.	Preclinical	Cancer, inflammatory diseases, colitis	[75]
GYY4137		National Uni. of Singapore	Preclinical	Inflammatory diseases, cancer, hypertension, arthritis	[82,107,123,152]
DAS/DADS			Preclinical	Cancer, arthritis	[148,149]
